# Factors associated with the preference of institutional delivery after antenatal care attendance in Northwest Ethiopia

**DOI:** 10.1186/s12913-019-4636-6

**Published:** 2019-11-07

**Authors:** Araya Mesfin Nigatu, Kassahun Alemu Gelaye

**Affiliations:** 1 Departmnet of Health Informatics, Institute of Public Health, College of Medicine and Health Sciences, University of Gondar, P.O.Box 196, Gondar, Ethiopia; 2 Department of Epidemiology and Biostatistics, Institute of Public Health, Dabat Health and Demographic Research Center, University of Gondar, P.O.Box 196, Gondar, Ethiopia

**Keywords:** Institutional delivery, ANC, Lay Gayint District, Northwest Ethiopia

## Abstract

**Background:**

Even though maternal mortality during the time of delivery can be prevented with proper medical care in the health facilities with skilled healthcare professionals, unexpectedly death is still high and is a persistent challenge for low-income countries. Therefore identifying factors affecting the preference of institutional delivery after antenatal care service attendance is a key intervention to reduce maternal morbidity and mortality.

**Method:**

A community-based cross-sectional study was conducted using face to face using interviewer-administered questionnaire from a total of 528 women who gave their last birth within 12 months prior to the study period who attended antenatal care (ANC) services. Descriptive statistics, bivariable and multivariable logistic regressions analysis were performed. Statistical significance was considered at *p* < 0.05 and odds ratio with 95% CI were calculated to examine factors associated with institutional delivery.

**Results:**

Of the 528 pregnant women attending ANC services, 250 (47.3%) gave birth in health facilities (95% CI: 43.2, 51.7%). Urban residence [AOR = 7.8, 95% CI: 4.1, 15.6], four or more ANC visits [AOR = 4.5, 95% CI: 1.6, 12.3], those who got health education on ANC [AOR = 2.9, 95% CI: 1.5, 5.6] and decision on place of delivery with her partner agreement [AOR = 3.3, 95% CI: 1.3, 8.7] were found to be contributing factors for the preference of institutional delivery.

**Conclusion:**

Institutional delivery was not adequate. Residence, number of antenatal care visits, health education, decisions making on a place of delivery and having awareness of the difference of place of delivery were contributing factors for the preference of institutional delivery.

## Background

Even though the global maternal mortality ratio declined by 44% from 385 deaths to 216 deaths per 100,000 live births (from 1990 to 2015), it is still a global critical challenge which didn’t show remarkable change from time to time [1]. Woman’s lifetime risk of maternal death is 1 in 4900 in high-income countries, versus 1 in 180 in low-income countries. Low-income countries take the major share of maternal deaths (99%); of these more than half occur in sub-Saharan Africa and almost one third occur in South Asia [2].

If a woman dies from complications of pregnancy and childbirth or becomes ill or disabled, the whole family’s health and wellbeing are often severely affected [3].

Though there are many contributing factors for high maternal mortality; causes related to pregnancy and childbirth are the major one including bleeding after delivery, unsafe abortion, pregnancy-induced hypertension, a complication from delivery, long duration of labor and infections shares the highest portion for mothers to lose their life [2].

Institutional delivery service utilization is one of the key and recognized intervention mechanism to decrease maternal death. Timely intervention warrants safe birth, minimize complications and maternal death by increasing the survival time of both mothers and newborns; however, most deliveries in low-income countries occur at home without the assistance of skilled birth attendants [4–6] which ultimately leads to lost their life. In high-income countries, skilled attendance is about 99.5% whereas in Africa below half (46.5%) [7] . According to Ethiopian Demographic and Health Survey (EDHS) 2016 report, 56% of pregnant mothers who attended more than four ANC visits gave birth at health facility assisted with skilled health professionals [8]. Regular ANC attendance is believed to guarantee healthier pregnancies and uneventful deliveries, and women who miss visits are considered at risk of poor pregnancy outcomes [9].

Therefore, identifying factors affecting institutional delivery after attending antenatal care is important and enables policy makers, partners, planners, and other health stakeholders to formulate appropriate strategies to increase institutional delivery rates and provide quality health care services.

## Methods

### Study design, period and settings

Community-based cross-sectional study was conducted to assess factors associated with the preference of institutional delivery after ANC visits in Lay Gayint district, Amhara National Regional State, northwest Ethiopia.

**Fig. 1:**
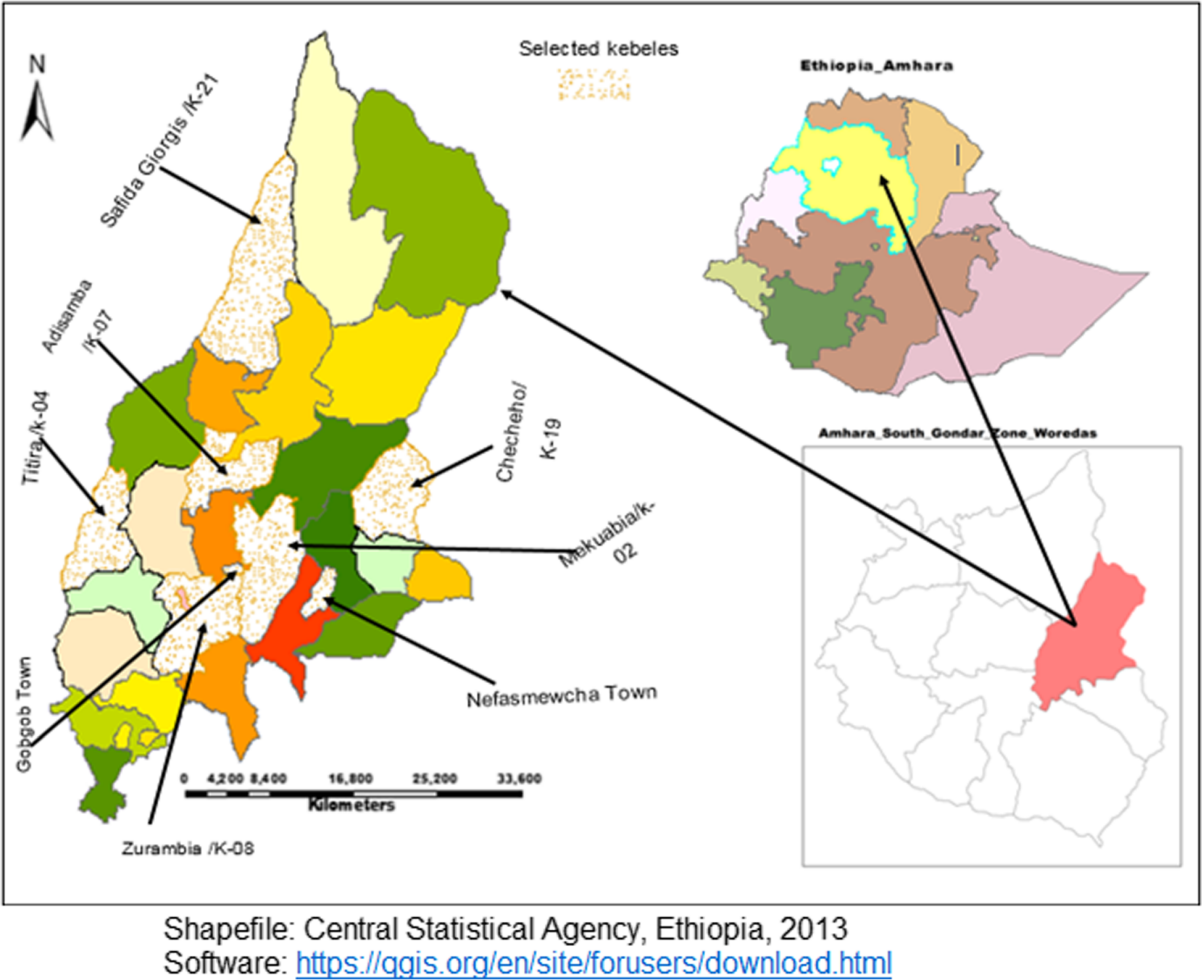
Location map of Lay Gayint district in South Gondar Administrative Zone

The study was conducted from February 15, 2016, to March 13, 2016. The district has a total of 29 *kebeles (*the smallest administrative units in Ethiopia*).* Of which, 25 are rural and the rest were urban. In the district 9 health centers (expected to give both curative and preventive services up to 25,000 population), 38 health posts (expected to give majorly preventive and very few curative services up to 5000 population) and 1 primary hospital (expected to perform preventive and advanced curative services than health centers up to 100,000 population) to the community (Fig. 1).

The district has an estimated population of 230,561. Of which 116,322(50.4%) were women; 31,511(13.7%) were urban inhabitants. Of the total female population women in a reproductive age constituted were about 54,956(47.2%) and the annual number of birth was expected to be 1852 [10].

### Sample size determinations and sampling procedures

The sample size was determined by using single population proportion formula with Z α/2 = 1.96, proportion of institutional delivery (*p* = 0.316) [11] and d (margin of error) = 5%. The final sample size after adding 10% non-response rate and design effect of 1.5 was 548. Eight kebeles (25% from the total 29 kebeles) were selected using simple random sampling technique. Of the selected kebles two of them were urban and the rest were rural. The calculated sample size was proportionally allocated to the selected kebeles. Finally, the study participants were selected by simple random sampling technique using their list as a sample frame then they were traced using their full address obtained from the ANC clinics who delivered for the last 12 months before the study period.

#### Operational definition

Institutional delivery was operationally defined as delivery in any health facility attended by skilled attendants. Skilled professionals were operationally defined as health personnel who were able to attend and respond uncomplicated and complicated labor at the health facility.

### Data collection procedures and quality control measures

Data were collected using interviewer-administered structured and pretested Amharic version questionnaire. The questionnaire was first developed in English and then translated to the Amharic language for appropriateness in approaching the study participants and then translated back to English by language experts to check its consistency. The questionnaire was comprised from socio-demographic, accessibility, behavioral and obstetric factors variables (Additional file 1: Questionnaire).

Two days training was given for data collectors and supervisors on the objective of the study, data collection procedures, data collecting tools, respondents approach, data confidentiality and respondents’ right prior to the data collection date. The completeness of the questionnaire was checked every other day by the supervisors and principal investigator.

### Statistical analysis

Data were entered, edited and cleaned using Epi-info version 7 and exported to SPSS version 20 for further statistical analysis. Descriptive statistics were used to summarize the data. The Logistic regression model was done to identify factors associated with the preference of institutional delivery.

Variables found to have an association with the dependent variable less than 0.2 *p*-value during variable analysis were entered into multivariable binary logistic regression using backward LR method for controlling the possible effects of confounders. Finally, the variables which had significant association were identified on the bases of odds ratio (OR), with 95% CI. The goodness of fit test was also checked using Hosmer-Lemeshow, the value was 77.3%.

### Ethics

Ethical clearance was obtained from the Institutional Review Board (IRB) of the University of Gondar. Permission letter to carry out the study was obtained from the Amhara National Regional State Health Bureau (ARSHB) and Lay Gayint District Health Office. Informed consent was obtained from each study participants after being told the purpose and procedures of the study. All responses were kept confidential and anonymous.

## Results

### Socio-demographic characteristics of mothers

In this study, a total of 528 women who had at least one ANC visit for their last pregnancy and gave birth were interviewed with a response rate of 96.4%. The mean age of the respondents was 28.12 years (SD ±6.1) and 152(28.82%) of the respondents were within 25 to 29 years of age category. Of the total, 143(27%) were from the urban residence, 484(91.7%) were married and 511(96.8%) were Orthodox Christians. In addition; 480 (91.7%) were housewives, 247(46.8%) were unable to read and write, 185(35.0%) were poor and 491(93%) lived within 5kms away from the nearest health facility (Table 1).

**Table 1:** Socio-demographic characteristics of respondents in Lay Gayint district, northwest Ethiopia, June 2016 (*n* = 528)

Variables	Frequency	Percent
Residence		
Urban	143	27.1
Rural	385	72.9
Age of respondents at present		
< 20	59	11.2
20–24	103	19.5
25–29	152	28.8
30–34	100	18.9
> =35	114	21.6
Marital status		
Married	484	91.7
Single	24	4.5
^a^Others	20	3.8
Occupation		
Housewife	480	87.1
Civil servant	20	3.8
Merchant	19	3.6
Student	15	2.8
Daily laborer	14	2.7
Education		
Unable to read and write	247	46.8
Primary (1–8)	140	26.5
Secondary (9–12)	77	14.6
Read and write	40	7.6
Higher (diploma & above)	24	4.5
Religion		
Orthodox	511	96.8
Muslim	14	2.7
Protestant	3	0.6
Wealth Index		
Poor	185	35
Medium	286	54.2
Rich	57	10.8
Distance from health facility		
< =5 km	491	93
5 km	37	7

^a^others = separated, widowed, divorced

### Place of delivery, delivery attendants and reasons for preferring institutional delivery

**Fig. 2:**
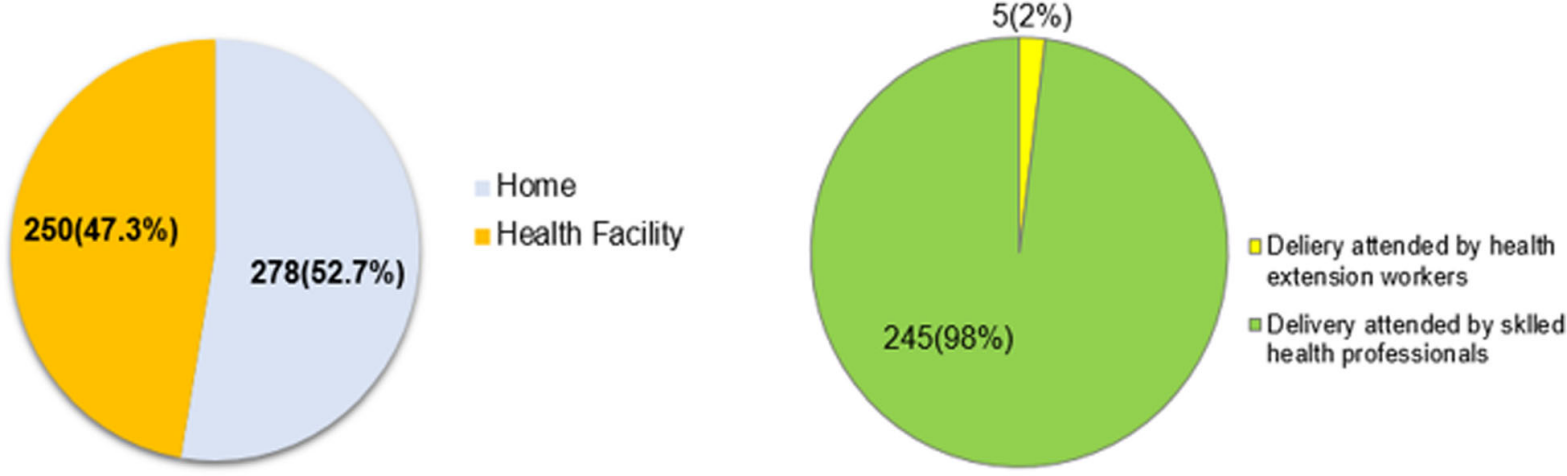
Place of delivery and delivery attendants in Lay Gayint district, northwest Ethiopia, June 2016

Of the total 528 mothers, 250(47.3%) gave birth at the health facility (95% CI: 43.2, 51.7%) and the majority 245(98%) were attended by skilled health professionals (Fig. 2).

**Fig. 3:**
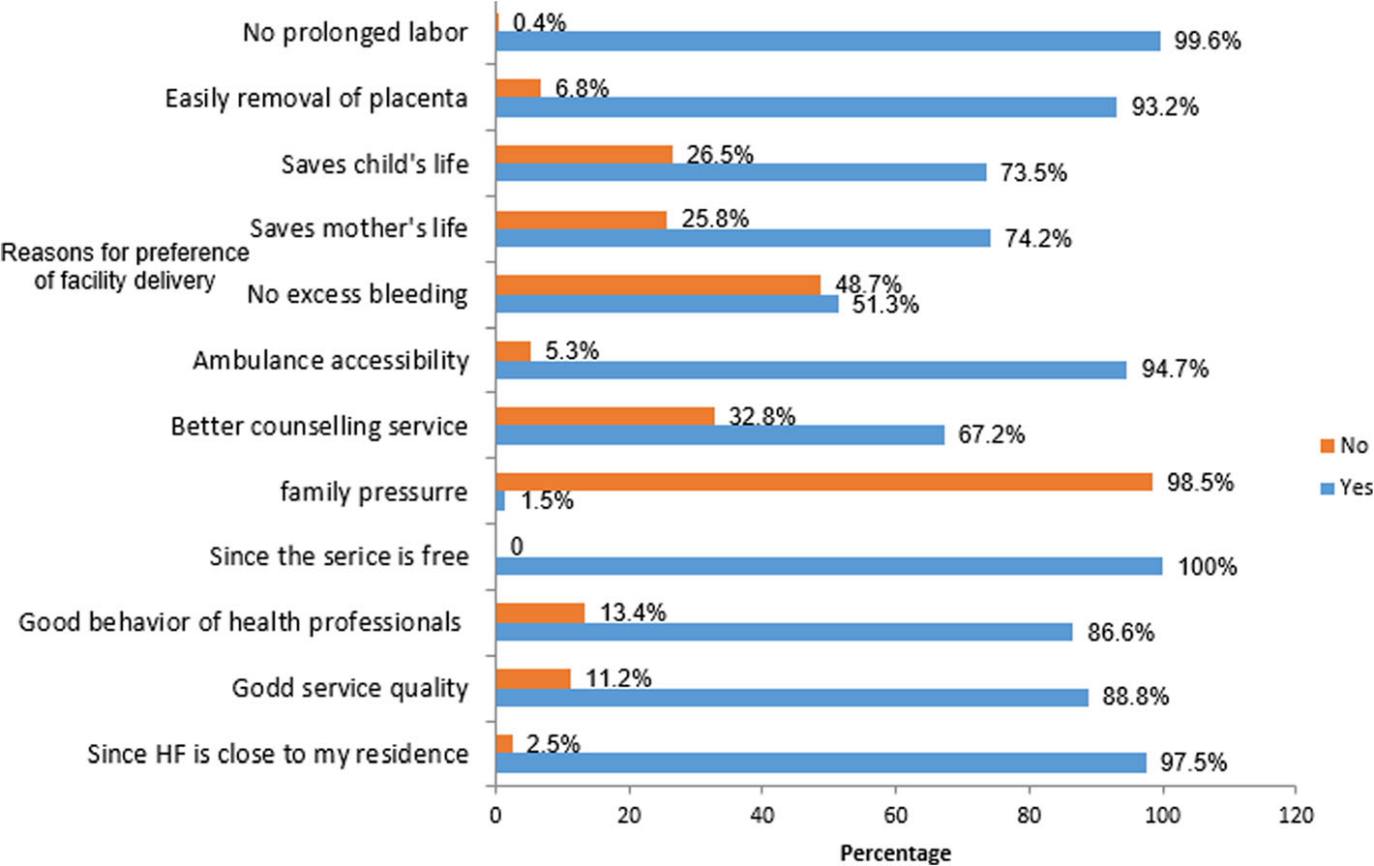
Reasons for the preference of institutional delivery in Lay Gayint district, northwest Ethiopia, June 2016

The major reasons for the preference of institutional delivery were: free availability of services (100%), short duration of labor (99.6%), nearby health facility (97.5%), availability of free ambulance service (94.7%), easily removal of placenta (93.2%), good service quality (88.8%), good approach of health care professionals (86.6%), save mother’s life (74.2%), save child’s life (73.5%), better counseling service (67.2%) and no excess bleeding (51.3%) (Fig. 3).

### Means of transportation to reach health facilities, obstetric characteristics of respondents and frequency of ANC visits

**Fig. 4:**
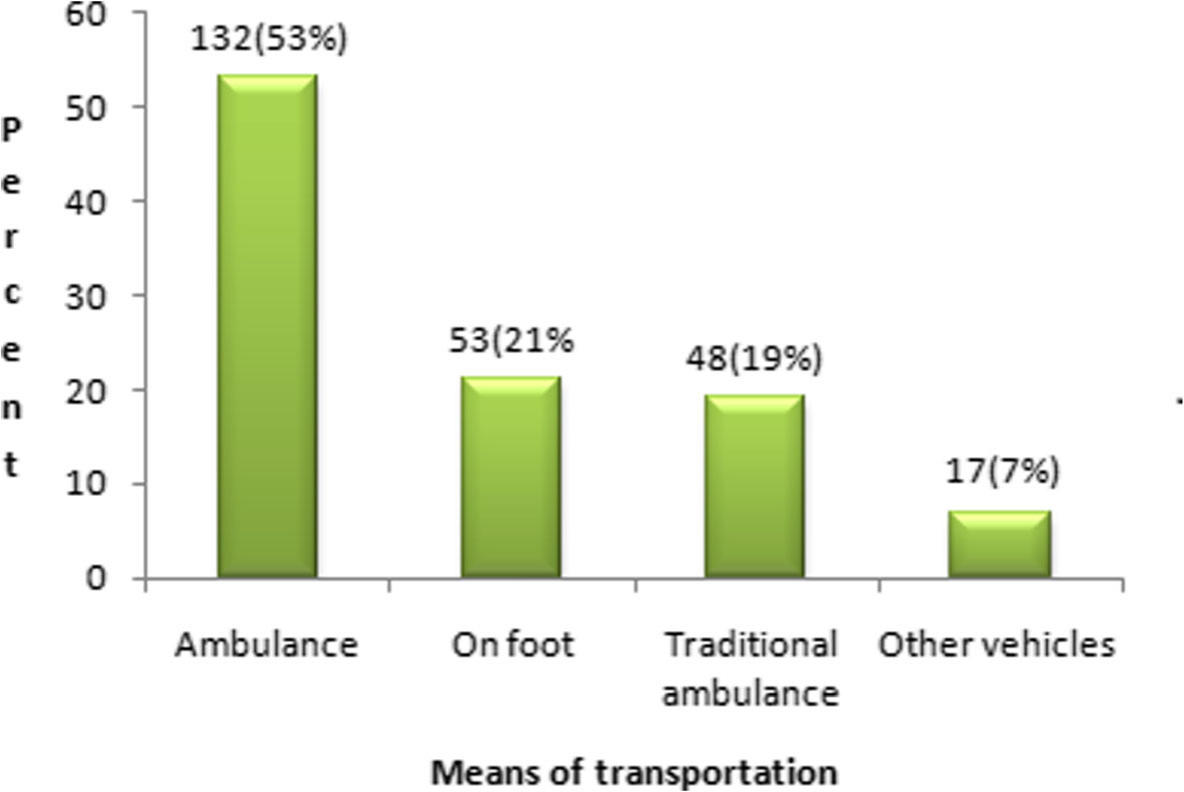
Means of transportations to reach health facilities in Lay Gayint district, northwest Ethiopia, June 2016

Among those who gave birth at health facilities (*n* = 250), 149(59.6%) used transport. Of these, slightly more than half 132(52.8%) used the ambulance to reach at health facilities (Fig. 4).

About 242(45.8%) of the respondents attended two to three antenatal care visits. Three hundred sixteen (59.8%) had two to four gravida and 320(60.6%) were having two to four parties (Table 2).

**Table 2:** Obstetric characteristics of respondents in Lay Gayint district, northwest Ethiopia, June 2016 (*n* = 528)

Variables	Frequency	Percent
Frequency of ANC visits		
One	79	15
Two to three	242	45.8
Four and above	207	39.2
Gravidity		
One	112	21
Two to four	316	59.8
Five and above	101	19.1
Parity		
One	111	21
Two to four	320	60.6
Five and above	97	18.4

### Health information and communication and behavioral characteristics of mothers

About 215 (40.7%) women received pregnancy and delivery related health information during their antenatal care visits at health facilities. About 284(53.8%) of women knew that the benefit of attending ANC for both the mothers and their child. Among those who got pregnancy and delivery related information, 413(78.2%) of them were decided their place of delivery together with their husband (Table 3).

**Table 3:** Behavioral and IEC characteristics of respondents in Lay Gayint district, northwest Ethiopia, June 2016

Variables	Frequency	Percent
Source of information ANC		
From health institution	215	40.7
From health extension workers	162	30.7
From radio/TV	74	14.0
From relatives and friends	77	14.6
Attending health education during each visit		
Yes	106	20.0
No/I don’t remember	422	80.0
Benefit of ANC		
Both the mother and the child	284	53.8
For child health	184	34.8
For maternal health	60	11.4
Decision on place of delivery		
Both of us	413	78.2
Myself	82	15.5
My husband	33	6.3
Is there a difference of giving birth at health facility instead of home?		
Yes	340	64.4
No/ I don’t know	188	35.6

### Factors associated with women’s institutional delivery

In this study residence, antenatal care visits, health education and decisions on a place of delivery were major contributing factors for institutional delivery at *p*-value < 0.05.

Urban residence mothers (AOR = 7.9, 95% CI: 4.1, 15.6) were eight times more likely to give birth at health facilities compared to rural counterparts.

Mothers who attended four and above antenatal care visit (AOR = 4.5, 95% CI: 1.6, 12.3) were four times more likely to deliver at health facility when compared to women who attended only one visit. Those who get pregnancy and delivery related information (AOR = 2.9, 95% CI: 1.5, 5.6) were three times more likely to deliver at health facilities compared to mothers who didn’t get any information during each ANC visits.

Mothers decision on a place of delivery done by an agreement with her husband (AOR = 3.3, 95% CI: 1.3, 8.7) were three times more likely to give birth at health institutions compared to the decision made by the mothers themselves only (Table 4).

**Table 4:** Bivariable and multivariable analysis of institutional delivery in Lay Gayint, northwest Ethiopia, June 2016 (*n* = 528)

Variables	Place of delivery		Crude OR	Adjusted OR
	Home (%)	HF (%)	(95% CI)	(95% CI)
Distance from the nearest health facility location				
< =5 km	252 (51.3)	239 (48.7)	2.24 (1.084, 4.637)	
> 5 km	26 (70.3)	11 (29.7)	1.00	
Residence				
Urban	29 (20.3)	114 (79.7)	7.19 (4.552, 11.379)	7.87 (4.086, 15.570)
Rural	249 (64.7)	136 (35.3)	1.00	1.00
Age				
< 20	31 (52.5)	28 (47.5)	1.38 (0.735, 2.611)	
20–24	46 (44.7)	57 (55.3)	1.9 (1.107, 3.261)	
25–29	71 (46.7)	81 (53.3)	1.75 (1.069, 2.863)	
30–34	61 (61)	39 (39)	0.98 (0.566, 1.698)	
> =35	69 (60.5)	45 (39.5)	1.00	
Respondent’s Education				
Unable to read and write	159 (64.4)	88 (35.6)	1.64 (0.834, 3.204)	
Read and write	21 (52.5)	19 (47.5)	1.52 (0.997, 2.321)	
Primary (1–8)	76 (54.3)	64 (45.7)	5.52 (3.089, 9.850)	
Secondary (9–12)	19 (24.7)	58 (75.3)	12.65 (3.669, 43.593)	
Higher (diploma & above)	3 (12.5)	21 (87.5)	1.00	
Source of information for antenatal care visit				
Health institution	119 (52.9)	96 (47.1)	2.15 (1.218, 3.800)	
HEW	82 (50.6)	80 (49.4)	2.60 (1.444, 4.686)	
Radio/TV	21 (28.4)	53 (71.6)	6.73 (3.302, 13.716)	
Friends & relatives	56 (72.7)	21 (27.3)	1.00	
Benefit of ANC				
Maternal health	50 (83.3)	10 (16.7)	1.87 (0.879, 3.960)	
Child health	134 (72.8)	50 (27.2)	10.11 (4.907, 20.816)	
Both the mother & child	94 (33.1)	190 (66.9)	1.00	
Total number of ANC visits				
1 visit	70 (88.6)	9 (11.4)	1.00	1.00
2–3 visits	147 (60.7)	95 (39.5)	5.03 (2.397, 10.540)	2.58 (0.963, 6.931)
≥ 4visit	61 (29.5)	146 (70.5)	18.62 (8.744, 39.632)	4.49 (1.636, 12.318)
Health Education				
Yes	34 (32.1)	72 (67.9)	2.9 (1.849, 4.558)	2.91 (1.504, 5.633)
No/I don’t know	244 (47.8)	178 (42.2)	1.00	1.00
A decision on a place of delivery				
My self	68 (82.9)	14 (17.1)	1.00	1.00
My husband	18 (54.5)	15 (45.5)	4.05 (1.654, 9.902)	3.71 (0.939, 14.672)
Both of us	192 (46.5)	221 (53.5)	5.59 (3.047, 10.257)	3.32 (1.269, 8.686)
Gravidity				
One	41 (36.9)	70 (63.1)	2.6 (1.495, 4.534)	
Two to four	176 (56)	140 (44)	1.21 (0.769, 1.915)	
5 and above	61 (60.4)	40 (39.6)	1.00	
Parity				
One	41 (36.9)	70 (63.1)	2.54 (1.451, 4.444)	
Two to four	179 (55.9)	141 (44.1)	1.71 (0.738, 1.859)	
5 and above	58 (59.8)	39 (40.2)	1.00	

*HF* Health facility, *1.00* Reference, Hosmer-Lemeshow Goodness of fit test = 0.773

## Discussion

Different findings showed that antenatal care increases the likelihood of an institutional delivery. In Ethiopia, 56% of births to mothers who attended more than four ANC visits were delivered in a health facility compared to 8% of births to mothers with no ANC visits.

In this study, 47.3% (95% CI: 43.2, 51.7%) of mothers delivered at the health facility. This finding appeared to be consistent with studies done in Urban Slum of Delhi (India) 47% [12] and Kenya 47% [13].

We found a higher proportion of facility delivery when compared to studies done in Ethiopian Munisa 12.3% [14], Cheha 31% [15] and Southwest Shoa Zone 28.6% [16]. The probable reasons for this might be, currently the government of Ethiopia is applying consistently free ANC, delivery and ambulance services at national levels in all health facilities which promote mother to seek healthcare services [17]. Eventually, these freely available services would boost the likelihood of women prefer to give birth at health institutions.

However, this finding was lower than studies done in Nigeria 60% [18], Ghana 79% [19], rural Chitwan (Nepal) 55% [20] and in Ethiopia, such as Bahir Dar town 78.8% [17] and Debre Markos Town 80.14% [21]. The possible justifications for this might be due to; better access of health facility with improved health infrastructure, quality health services, having better educational status, better media exposure to access health information, easy access of transportation which enforces pregnant mothers to give birth at health facilities.

The multivariable analysis identified different factors that influence the preference of institutional delivery. The odds of women from the urban residence were 7.87 times more likely to give birth at health institutions when compared to rural residences. This finding appeared to be similar with studies done in Ethiopia districts such as Wolisso, Wonchi and Goro [16] and other study done in Ghana as well [22]. The possible explanation for this might be due to; urban women might have got better information access regarding the importance of facility delivery and healthcare service with senior health care professionals (nurses, midwives and general practitioners) when compared to rural kebele women. In addition, the media exposure promoting good health services have been widely available in urban areas than rural residents which might influence the mother to escape out from outdated practices.

The number of antenatal care visits had also a significant statistical association with the preference of institutional delivery. Women who had four and above ANC visit were 4.5 times more likely to give birth at health facility when compared to those who attended only one visit. This finding is consistent with other studies done in different areas of Ethiopia districts such as Fogera [11], Sekela [23], Akansha Guagusa [24] and abroad in Tanzania [25]. This may be due to; better number of contact with the skilled provider during pregnancies gave chance for the women to acquire more information about the danger sign during pregnancy and the importance of facility delivery.

The odds of mothers who received any ANC services and delivery related health education were 2.91 times more likely to give birth at health institution when compared to those who didn’t receive any. These finding is consistent with studies done in northern Bangladesh [26] and Biharamulo district Tanzania [27]. The possible reason for this might be due to the fact that; getting more information during their ANC visits will help them to identify danger signs of pregnancy and its bad consequence when giving birth took place at home without skilled professionals which in turn them to prefer health facility delivery.

Decision made jointly by the mother and her husband on a place of delivery had also the significant statistical association with institutional delivery. Mothers who decided their place of delivery together with their husband were 3.32 times more likely to give birth at health facility when compared to the decision made by the mother only. This finding is similar to other studies conducted in Debre Markos Town (Ethiopia) [21] and Biharamulo district (Tanzania) [27]. The possible explanation for this might be; when communication is made between the wife and her partner, in fact, they will reach a successful agreement and ultimately made the right decision. In most African countries husbands share a higher percentage level of place of delivery i.e. husband’s explicit support for institutional delivery influences the chances of a child being born at a health facility.

Finally, respondents’ with educational level, the benefit of antenatal care visit, parity, gravidity, age, source of information for antenatal care and distance from the nearest health facility were not significantly associated with institutional delivery in the multivariable analysis.

Institutional delivery in an appropriate setting is a life-saving method that can also help mothers to reduce the risk of complications which may cause death or illness. Due to the cross-sectional nature the study, there are some limitations like difficult to avoid recall bias, identifying the cause-effect relationship and exploring behavioral determinants clearly. Therefore, conducting research using qualitative design might be appropriate to explore behavioral factors.

## Conclusion

In conclusion, preference of institutional delivery assisted by health care professionals even after attending antenatal care was low. The study also identified factors such as residence, number of antenatal care visits, health education, decisions making on a place of delivery and having awareness of the difference of place of delivery were significantly associated with preference of institutional delivery. Therefore, empowering women, awareness creation, promoting uptake of antenatal care visits and providing consistent free ambulance services are recommended interventions to increase the preference of institutional delivery.

## Supplementary information

**Additional file 1.** English version questionnaire used to assess factors associated with the preference of institutional delivery after antenatal care attendance.

## Data Availability

All relevant data are in the manuscript. However, the minimal data underlying all the findings in the manuscript will be available upon request by contacting the corresponding author.

## Abbreviations

ANC: Antenatal Care
ARSHB: Amhara Regional State Health Bureau
CSA: Central Statistical Agency
EDHS: Ethiopian Demographic Health Survey
IRB: Institute Review Committee
