# Supplementary material for: Factors associated with the preference of institutional delivery after antenatal care attendance in Northwest Ethiopia
Source: BMC Health Serv Res. 2019 Nov 7;19:810. doi: 10.1186/s12913-019-4636-6 (PMC6836405; doi:10.1186/s12913-019-4636-6)
Supplement: Supplementary file 1 — Additional file 1. English version questionnaire used to assess factors associated with the preference of institutional delivery after antenatal care attendance. [file 12913_2019_4636_MOESM1_ESM.docx]

## **Annex D: Questionnaire –English version**

| **S.No** | **Question** | **Response** | **Skip** |
| --- | --- | --- | --- |
| 101 | Religious | 1. Orthodox 3. Catholic 2. Muslim 4. Protestant |  |
| 102 | Residence | 1. Urban 2. Rural |  |
| 103 | Age in year at present | (_______) |  |
| 104 | Marital status | 1. Married 4. Divorced 2. Single 5. Widowed 3. Separated |  |
| 105 | Respondent’s occupation | 1. House wife 5. Student 2. Civil servant 6. Merchant 3. Farmer 7. Others specify--- 4. Daily laborer |  |
| 106 | If married what is the main occupation of your husband? | 1. Farmer 5. Driver 2. Daily laborer 6. Student 3. Merchant 7. Others specify---- 4. Civil servant |  |
| 107 | Respondent’s educational status | 1. Illiterate 4. Secondary (9-12) 2. Read and write 5. Higher (diploma 3. Primary (1-8) and above |  |
| 108 | If married, husband’s educational status | 1. Illiterate 2. Read and write 3. Primary (1-8) 4. Secondary (9-12) 5. Higher (diploma and above) |  |

**Part i: Questions related to socio-demographic characteristics**

**Part ii: Questionnaires on Wealth Index**

| **S.No** | **Question** | **Response** | **Skip** |
| --- | --- | --- | --- |
| 201 | Does your house hold have | - Electric city? - Radio? - Television? - Mobile telephone? - Fixed line telephone? - Refrigerator? - A bed with cotton? - Electric mitad? - Kerosene lamp |  |
| 202 | What type of fuel does your HH mainly used for cooking? (more than one answer is possible) | 1. Electricity 2. Biogas 3. Kerosene 4. Charcoal 5. Animal dung/wood |  |
| 203 | Where cooking is usually done in your house hold? | 1. In the house 2. In a separate building 3. Out doors 4. In a separate room /kitchen/ |  |
| 204 | Main material of the house floor | 1. Earth/sand 3. Cement 2. Dung 4. Ceramic tile |  |
| 205 | Main material of the roof | 1. Wood planks with grass 2. Wood planks with thin 3. Cement/concert |  |
| 206 | Main material of the external walls? | 1. Cement 2. Wood 3. Bricks |  |
| 207 | How many rooms in the house hold are used for sleeping? | (____________) in number |  |
| 208 | Ownership of the house? | 1. Private 4. Family 2. Kebele 5. Transfer 3. Rent 6. Others specify--- |  |
| 209 | Does any member of the HH own | - Bicycle? - Motor cycle? - Car/truck? - Animal drown cart? - Bajaj? |  |
| 210 | Does any member this HH own any agricultural land? | 1-yes  2-No | Skip to Q 213 |
| 211 | How many of agricultural land do members of this HH own? | (____________) in hector /Rope |  |
| 212 | Ownership of the agricultural land | 1. Own 2. Rent |  |
| 213 | Does this HH own livestock, herds, other farm animals or poultry? | 1-yes  2-No | Skip to Q 215 |
| 214 | Does your house hold have | - Cows? - Bulls? - Oxen? - Horses? - Donkeys? - Goats? - Sheep? - Chicken? - Beehives? |  |
| 215 | Does any member of this HH have a bank account? | 1. Yes 2. No |  |

**ART iii: Antenatal Care Information**

| **S.No** | **Question** | **Response** | **Skip to/Code** |
| --- | --- | --- | --- |
| 301 | Where did you hear about the source of ANC services? | 1. Health institutions 2. Radio/TV 3. Relatives and friends 4. Health extension workers 5. Other specify--------- |  |
| 302 | What did you think about the benefit of ANC? | 1. Maternal health 3. Both of us 2. Child health 4. Others ----- |  |
| 303 | What was the total number of visits? | 1. 1^st^ visit 3. 3^rd^ visit 2. 2^nd^ visit 4. 4 and above visit |  |
| 304 | To which health institution did you go for your ANC Check-ups? | 1. Health post 3. Primary hospital 2. Health center 4. Private clinic |  |
| 305 | Was health education given for each ANC visit? | 1. Yes 2. No 3. Don’t know |  |

**Part iv: Women’s place of delivery and reason for their choice**

| **S.No** | **Question** | **Response** | **Skip to/Code** |
| --- | --- | --- | --- |
| 401 | Where did you prefer to deliver your child? | 1. Health facility 2. At home | skip to Q407 |
| 402 | If health facility, what type of health facility you have visited for your delivery? | 1. Health post 3. Primary hospital 2. Health center 4. Private clinics |  |
| 403 | If at health facility, why did you prefer to give birth at health facility? (More than one answer is possible) | 1. Close where I live 2. High quality of delivery service 3. Good approach of health workers 4. No fee for the service 5. Family pressure 6. Received health education 7. Availability of quick ambulance service 8. Others specify____ |  |
| 404 | What means of transportation did you use? | 1. On foot 3. Other vehicles 2. Ambulance 4. Traditional ambulance |  |
| 405 | If your answer was on foot or traditional ambulance why? | 1. There is no road for vehicles 2. I don’t have money to pay 3. My residence is close to HF 4. Ambulance service delay 5. No ambulance service |  |
| 406 | Who assisted you at the health facility during delivery? | 1. Health Extension workers 2. Skilled health professionals 3. I don’t remember |  |
| 407 | If at home who assisted you? | 1. Mother 4. TBA 2. Neighbor 5. HEWs 3. Mother-in-low 6. Others specify---- |  |
| 408 | What is the reason to give birth at home? (more than one answer is possible) | 1. Labor was not too long 2. Transportation problem 3. Distance from HF is far 4. Poor service of HF 5. Feel sham 6. I was sick during my labor 7. Other’s specify_____ |  |
| 409 | Who decided your place of delivery? | 1. My self 3. Both of us 2. My husband 4. Others specify |  |
| 410 | Difference of giving birth at health facility instead of home? | 1. Yes 2. No 3. Don’t know | If no Skip to 412 |
| 411 | If you think health facility was better why? (More than one answer possible) | 1. Clean 2. No bleeding 3. Save mothers life 4. Saves child life 5. No retain placenta 6. Shorter labor |  |
| 412 | If you think home is better why? (More than one answer is possible) | 1. No need of transport 2. No need of extra cost 3. There is privacy 4. Better home ceremony 5. Other specify_____ |  |
| 413 | Where was the choice of your husband to your place of delivery? | 1. Health institution 2. Home |  |

**v. Obstetric care history**

| 501 | Gravidity/total number of pregnancy | (__________) in numbers |  |
| --- | --- | --- | --- |
| 502 | Parity/total number of births | (__________) in numbers |  |

This is the end of our interview. Thank you very much for your time. We appreciate your help!
